# Pathogens Spillover from Honey Bees to Other Arthropods

**DOI:** 10.3390/pathogens10081044

**Published:** 2021-08-17

**Authors:** Antonio Nanetti, Laura Bortolotti, Giovanni Cilia

**Affiliations:** Council for Agricultural Research and Agricultural Economics Analysis, Centre for Agriculture and Environment Research (CREA-AA), Via di Saliceto 80, 40128 Bologna, Italy; antonio.nanetti@crea.gov.it (A.N.); giovanni.cilia@crea.gov.it (G.C.)

**Keywords:** spillover, inter-species transmission, honey bee diseases, pathogens, virus, bacteria, microsporidia, *Nosema*, trypanosomatids, wild bees, arthropods, Hymenoptera

## Abstract

Honey bees, and pollinators in general, play a major role in the health of ecosystems. There is a consensus about the steady decrease in pollinator populations, which raises global ecological concern. Several drivers are implicated in this threat. Among them, honey bee pathogens are transmitted to other arthropods populations, including wild and managed pollinators. The western honey bee, *Apis mellifera*, is quasi-globally spread. This successful species acted as and, in some cases, became a maintenance host for pathogens. This systematic review collects and summarizes spillover cases having in common *Apis mellifera* as the mainteinance host and some of its pathogens. The reports are grouped by final host species and condition, year, and geographic area of detection and the co-occurrence in the same host. A total of eighty-one articles in the time frame 1960–2021 were included. The reported spillover cases cover a wide range of hymenopteran host species, generally living in close contact with or sharing the same environmental resources as the honey bees. They also involve non-hymenopteran arthropods, like spiders and roaches, which are either likely or unlikely to live in close proximity to honey bees. Specific studies should consider host-dependent pathogen modifications and effects on involved host species. Both the plasticity of bee pathogens and the ecological consequences of spillover suggest a holistic approach to bee health and the implementation of a One Health approach.

## 1. Introduction

Interspecific transmission may occur from a definite maintenance host (aka “reservoir”) to an incidental or non-maintenance species (aka “spillover host”). Spillover cases are crucial to pathogen dynamics [1,2].

In a single-host scenario, reservoirs are sufficient and pathogen replication does not need other host species [3]. The basic reproduction number (R0) defines the frequency of new cases originating from each primary event, where R0 = 1 is the threshold between declining infections (R0 < 1) and pathogen persistence within the population by intra-specific transmission (R0 > 1) [4]. When multiple host species are involved, the presence of new maintenance or incidental hosts may result in an increased pathogen transmission [1]. In this case, R0 >> 0 denotes multi-host pathogen scenarios that may be respectively true or apparent, depending on the high or low interspecies transmission. When the R0 is between 0 and 1, the event is called “apparent multi-host pathogen”, while “true multi-host pathogen” indicates an event in which there are two different maintenance hosts and the occurrence of interspecies transmission is higher than 1 [5].

Strictly speaking, spillover only occurs when the recipient species is characterized by R0 ≈ 0 [5]. However, in this review, we follow the use of the term *sensu lato*, commonly indicating a multifaceted range of host shift events [2].

Pollinators are crucial to the generation of crops contributing to the human diet [6]. These agroecosystem service is provided by a range of different species, including honey bees, wild bees, wasps, hoverflies, and butterflies [7,8,9,10]. However, different factors contribute to a decline of pollinating entomofauna, in terms of population size, biodiversity, abundance and distribution [11,12,13,14,15,16,17,18,19].

Pathogens and parasites are deemed drivers of this decline, together with other factors including pesticides and global warming. Nonetheless, the global picture is certainly far from complete, since data may misrepresent the actual distribution and gaps remain in our understanding of both epidemiological features and invasion dynamics of many pathogens [12,20,21,22,23,24,25]. *Apis mellifera* is known to share pathogens with bumblebee species, including viruses, bacteria, fungi and protozoa [26,27,28,29,30,31,32,33,34]. After acting as incidental hosts, western honey bees may become the primary maintenance host, as occurred in the cases of *Nosema ceranae*, *Crithidia bombi*, and *Apicystis bombi* [35]. Pathogens may also genetically adapt to a range of new species [12,13,23,30,36], acting as incidental or maintenance hosts [37,38,39].

Interspecific transmission to arthropods sharing the same environment as honey bees may occur orofecally, via direct contact and by pollen contamination [38]. Besides, infected foragers may contaminate pollen, nectar and floral organs with pathogens [40,41,42,43,44]. Spillover could also involve species not expected to come into direct contact with the bees. Wasps predating infected bees [45,46,47,48] and cannibalizing their carcasses [49,50,51] are likely to become contaminated with pathogens.

Honey bees and other insects have natural immune defense systems against bacteria, protozoa, mites, and viruses. They include antimicrobial peptides like apidaecin, defensin, abaecin, hymenoptaecin, and lysozyme, which are regulated by the immune pathways Toll, IMD, JAK/STAT and JNK [52]. Those defenses are challenged by insecticides and other pesticides used in modern agriculture [53].

Spillover events are difficult to prove. Indeed, viral infection and replication in new hosts, which may not develop under artificial conditions, can occur in nature [36,54,55]. The increasing number of reports about honey bee pathogens found in new hosts contributes to depict a scenario including one reservoir species and multiple spillover events. Indeed, population studies might elucidate those aspects [56] which, in the specific case of wild bees, are complicated by the peculiar characteristics of those species [17,57,58]. This makes spillover routes generally unknown and undetermined [59], albeit each report deserves further research to illustrate thoroughly their respective epidemiological scenarios.

This systematic review is intended to collect, group, and summarize the spillover cases *sensu lato* reported by the literature and involving honey bee pathogens. Other arthropods were also considered as alternative hosts. The spillover cases are grouped by: (i) host species, condition and stage, (ii) geographical region and year of the report, and (iii) co-occurrence in the same host.

## 2. Results

**Figure 1:**
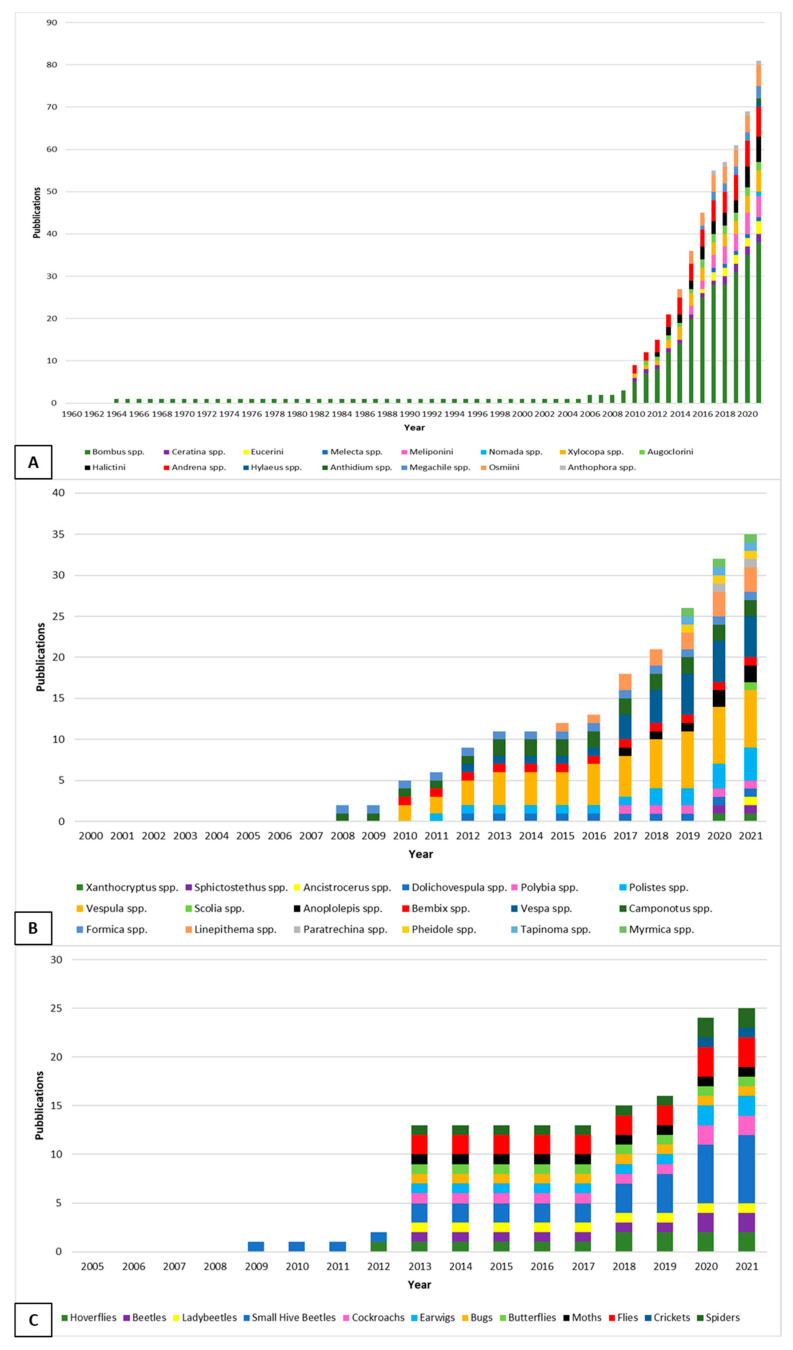
Cumulative number of spillover studies of honey bee pathogens available in the literature between 1960 and 2021 involving other bees (**A**), non-bee Hymenoptera (**B**), and other arthropods (**C**).

In total, from 1960 to 2021, 81 studies investigated spillover cases of honey bee pathogens to wild and/or managed arthropods (Figure 1). Some of the studies considered more than one species. In detail, they considered the spillover to other bee species (Supplementary Table S1), other Hymenoptera (Supplementary Table S1) and other arthropods (Supplementary Table S2).

As shown in Figure 1A, the first article about spillover of honey bee pathogens to other bees was published in 1964, but the number of articles on this topic steadily increased from the year 2020, likely due to the quick development of molecular genetic tools for pathogen detection. Considering other hymenopteran species, the first detection of spillover cases dates back to 2008, with a rapid increase of cases in the following years (Figure 1B). The first spillover case to other arthropods was assessed in 2009, but later the frequency increased, covering a wide range of species (Figure 1C).

**Figure 2:**
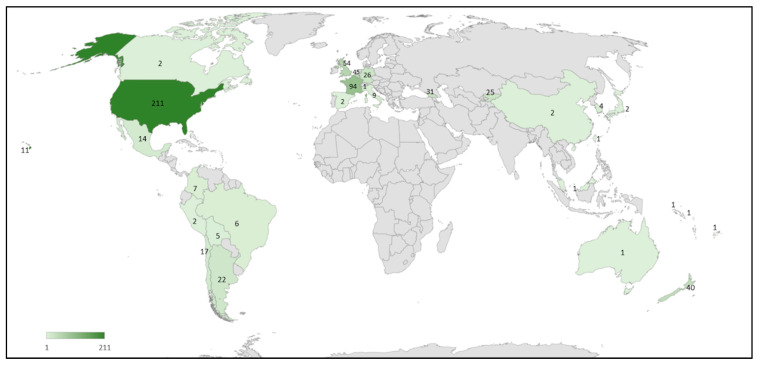
Geographical distribution of the honey bee pathogen spillover studies reported in the literature. The number of published cases is indicated for each country and highlighted by different shades of green.

The geographical distribution of spillover studies present in the literature (Figure 2) shows a high number of studies in both North and South America, Europe and New Zealand, whereas the reports from other countries were less frequent. 

**Figure 3:**
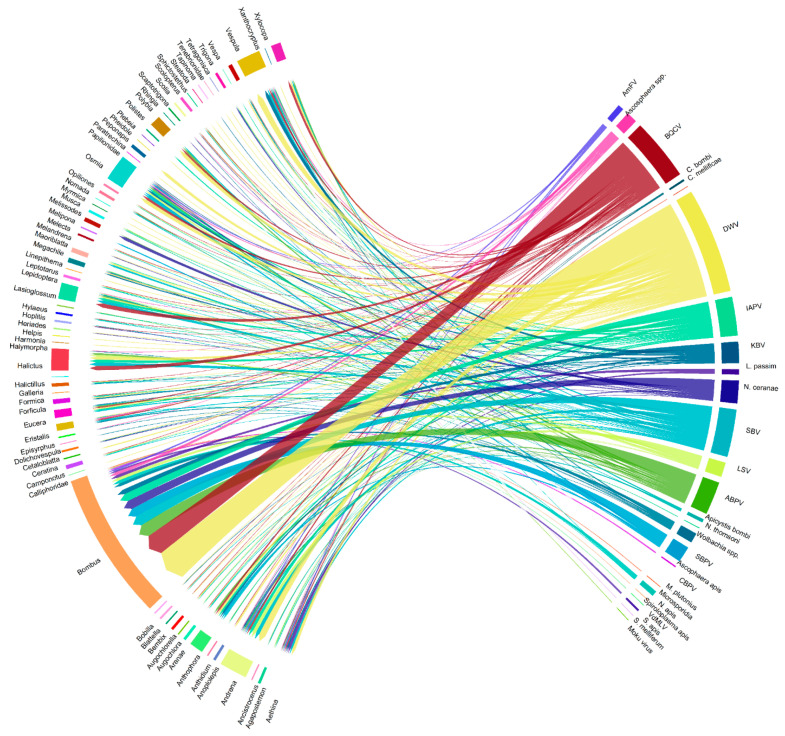
Visual schematization of honey bee pathogen spillover to alternative host arthropods reported in the literature. Different colors denote distinct pathogens or host genera. Legend: ABPV: Acute Bee Paralysis Virus; IAPV: Israeli Acute Paralysis Virus; BQCV: Black Queen Cell Virus; SBV: Sacbrood Virus; DWV: Deforming Wing Virus; LSV; Lake Sinai Virus; *Am*FV: *Apis mellifera* Filamentous Virus; KBV: Kashmir Bee Virus; SBPV: Slow Bee Paralysis Virus; CBPV: Chronic Bee Paralysis Virus; VdMLV: *Varroa destructor* Macula-like Virus.

**Figure 4:**
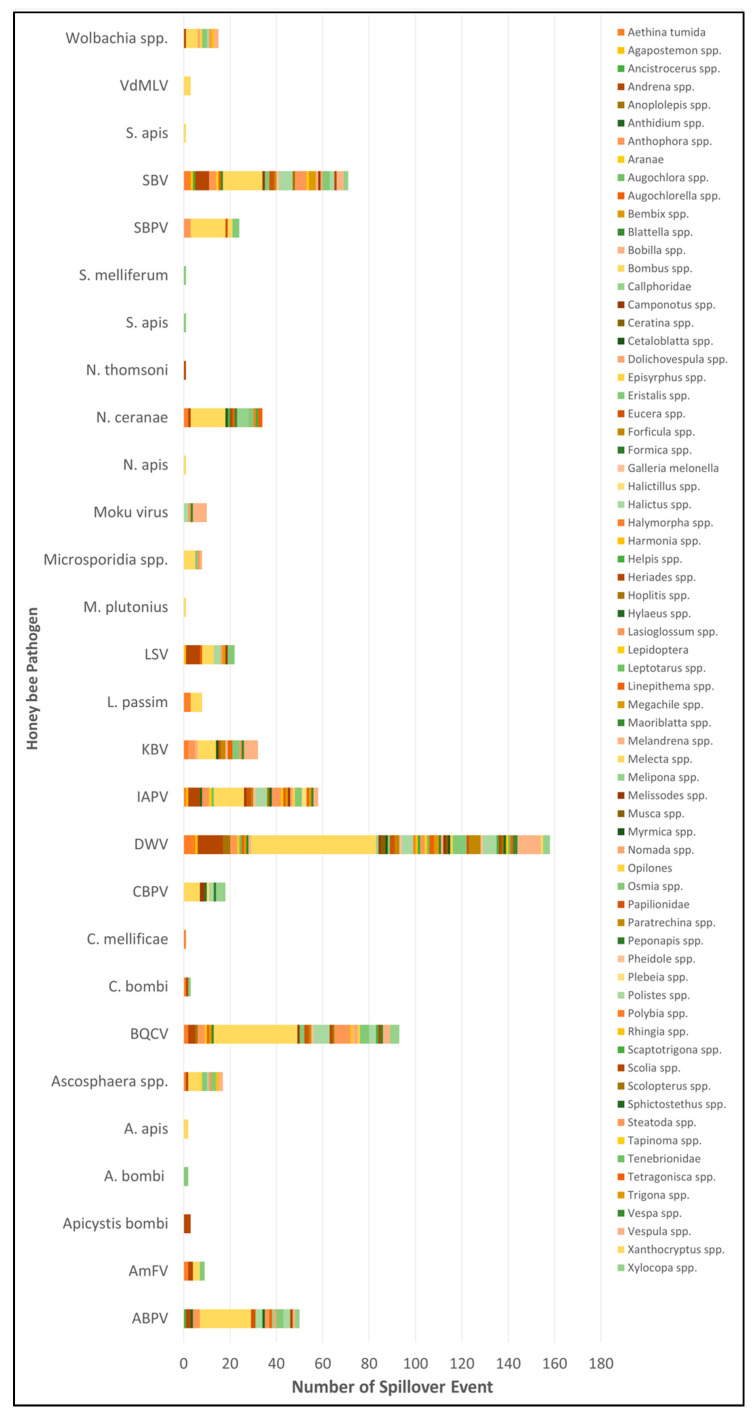
Frequency of spillover events involving single honey bee pathogens and the range of arthropods found infected with them. Different colors denote distinct host groups. Legend: ABPV: Acute Bee Paralysis Virus; IAPV: Israeli Acute Paralysis Virus; BQCV: Black Queen Cell Virus; SBV: Sacbrood Virus; DWV: Deforming Wing Virus; LSV; Lake Sinai Virus; *Am*FV: *Apis mellifera* Filamentous Virus; KBV: Kashmir Bee Virus; SBPV: Slow Bee Paralysis Virus; CBPV: Chronic Bee Paralysis Virus; VdMLV: *Varroa destructor* Macula-like Virus.

Figure 3 and Figure 4 summarize the spillover cases for each honey bee pathogen in relation to arthropods groups. In events encompassing at least 20 spillover cases, DWV was the most frequently detected (158 cases). BQCV, SBV, IAPV, ABPV, KBV, *N. ceranae*, SBPV and LSV resulted implicated with progressively decreasing frequency.

The chord graph (Figure 3) shows all spillover cases described in this review, evaluating the relationship to the investigated arthropod genus. Additionally, Figure 4 highlights the reported frequency of honey bee pathogens in the investigated arthropod communities, to emphazise their plasticity to the host.

**Figure 5:**
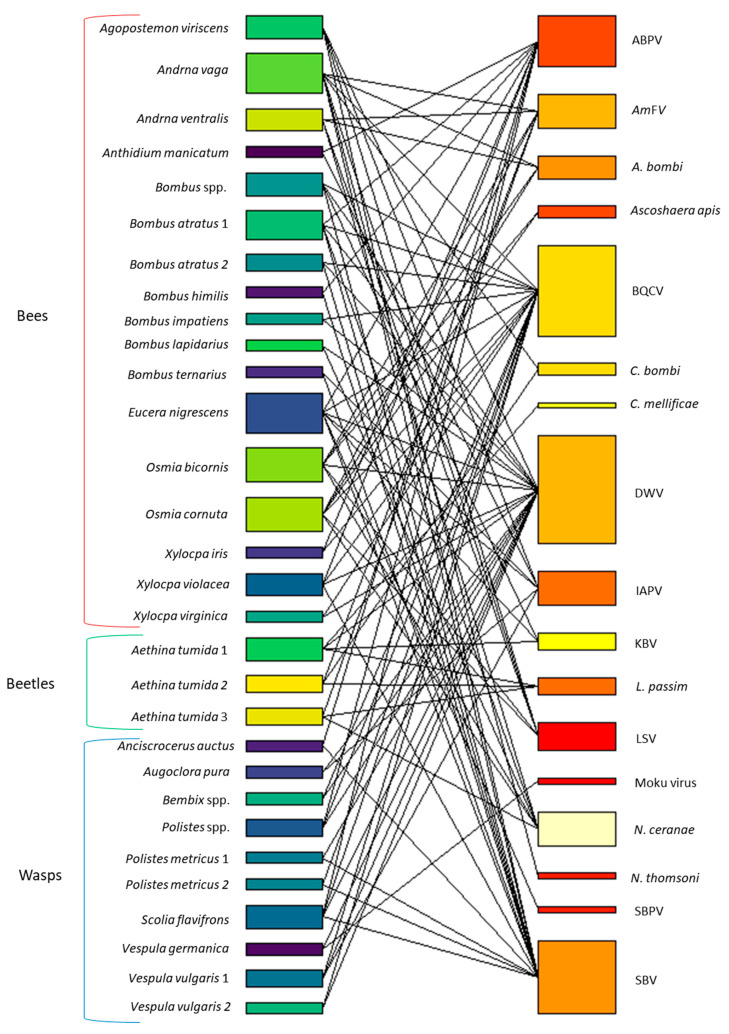
Co-occurrence of honey bee pathogens in individual hosts. These are grouped as bees, beetles, and wasps. Box size is indicative of the frequency. Legend: ABPV: Acute Bee Paralysis Virus; IAPV: Israeli Acute Paralysis Virus; BQCV: Black Queen Cell Virus; SBV: Sacbrood Virus; DWV: Deforming Wing Virus; LSV; Lake Sinai Virus; *Am*FV: *Apis mellifera* Filamentous Virus; KBV: Kashmir Bee Virus; SBPV: Slow Bee Paralysis Virus; CBPV: Chronic Bee Paralysis Virus; VdMLV: *Varroa destructor* Macula-like Virus.

Some individuals were found infected with multiple honey bee pathogens (Figure 5). The highest incidence of coinfections was found in bumblebees, followed by mason bees, mining bees and the honey bee pest *Aethina tumida*. A high number of co-infections was reported for *Eucera nigrescens, Osmia bicornis* and *Osmia cornuta*, for which 6 pathogens were found in the same individuals. Besides, the most abundant coinfecting pathogens able to co-infect the arthropods hosts were DWV, BQCV, SBV, ABPV and *N. ceranae*.

## 3. Discussion

The results of this systematic review highlights that the case history of spillover events involving honey bee pathogens increased over the past six decades. This is consistent with the growing interest of the scientific community in understanding the underlying factors [12,20,22,54]. The higher incidence of spillover cases recorded in Europe, New Zealand, and the Americas may reflect their advances in research and apiculture compared to other regions [60,61,62,63,64,65,66,67].

Viruses vectored by *V. destructor* (DWV, KBV, and IAPV) and quasi-ubiquitous pathogens (BQCV, SBV and *N. ceranae*) were among the most frequently reported cases.

Bumblebees, mason bees and leafcutter bees were the species in which the spillover was studied more intensely, possibly because of their use in crops and fruit pollination. The fact that some of the surveys were carried out on arthropods ranging freely in the same environment as the managed honey bees is indicative of a pathogen circulation in their common environment. Despite honey bee pathogens were detected in other arthropods, symptoms and other effects on the alternative host populations remain unknown—except for some publications reporting individual bumblebees with crippled wings and scoring positive to DWV [68,69].

The importance of investigating the spillover of honey bee pathogens is also indicated by the discovery of active coinfections in wild hymenopteran individuals. As for the honey bees [30,70,71], multiple infections were found in wild bees, wasps and *Aethina tumida* individuals, which shows the importance of other arthropods as incidental hosts. The multiple infections that were identified (Figure 5 and Supplementary Table S1) have both the effect to increase the circulation of pathogens within the arthropod communities, and to recirculate them to the managed honey bee colonies, so generating damage at individual and colony levels.

All of these aspects, including their modifications and effects encompass the implementation of a One Health approach to bee health [72,73]. The health of managed honey bees is dependent on the health of wild bees and other arthropods, and vice versa. This approach is essential to provide suitable ecosystems to pollinators and other arthropods contributing to human livelihoods and environmental health, and for understanding the eco-immunology to prevent the transmission of pathogens and pests, thereby limiting damages in managed and wild insect populations [73,74,75]. Therefore, the circulation/re-circulation and the possible impact of honey bee pathogens to the arthropod communities are crucial to build the basis for the One Health approach to the bee health. Here we provide a brief discussion of each of the honey bee pathogens reported in Supplementary Tables S1 and S2, in relation to their spillover hosts.

### 3.1. Viruses

#### 3.1.1. Deformed Wing Virus (DWV)

DWV is a non-enveloped ssRNA (+) virus belonging to *Iflavirus* genus within the *Picornaviridae* family [76]. The DWV is a pathogen including three distinct genomotypes: A, B and C [77,78].

The DWV is probably the most known, spread, prevalent, and studied honey bee pathogen, often associated to *V. destructor* [79]. The DWV can be asymptomatically replicated in *V. destructor* mites [80].

The impact of DWV on honey bees leads to increased interspecific transmission, reaching several species of hymenopterans and other arthropods (Supplementary Tables S1 and S2).

The virus was identified not only in species living in close contact with the honey bees, like *A. tumida*, *G. mellonella, Vespa* spp. [39,45,47,81,82], but also in *Apis* and non-*Apis* species that may act as incidental hosts [38,39]. DWV was found in naturally and artificially infected asymptomatic arthropods [38,39,54,59,79], although some commercial and wild *B. terrestris* and *B. pascuorum* individuals were found with crippled wings [68,69]. Besides, artificial infection experiments highlighted that DWV reduced the individual lifespan in some *Bombus* species [40,83,84,85] or generate reinfection in the honey bees [38,39,81,82,86].

#### 3.1.2. Kashmir Bee Virus (KBV)

KBV is a non-enveloped ssRNA (+) virus belonging to the *Cripavirus* genus within the *Dicistroviridae* family [28,81,87]. The genome of KBV is strictly related to ABPV (Acute Bee Paralysis Virus) and IAPV (Israeli Acute Paralysis Virus) [88,89,90].

Although the virus is considered endemic in America and New Zealand, it has been rarely reported in other regions, both in honey bees and other arthropods (Supplementary Tables S1 and S2).

KBV was found in various Hymenoptera species, like *Bombus* spp. [38,91,92,93], *Eucera* spp., *Anthophora* spp., *Osmia* spp. [38], wasps, hornets [46,91,94,95], and ants [49,91]. It was also detected in *A. tumida* [39,81], *Galleria melonella*, earwigs, roaches and crickets [39,91].

#### 3.1.3. Acute Bee Paralysis Virus (ABPV)

ABPV is a non-enveloped virus and widespread ssRNA (+) virus belonging to *Apavirus* genus within the *Dicistroviridae* family [28,96]. As reported above, ABPV is genetically linked to KBV and IAPV [88]. ABPV was detected in *V. destructor*, where is is reported incapable to replicate [97,98]. ABPV spillover is not recent (Figure 1 and Supplementary Table S1) as in 1964 various *Bombus* species were found infected in the United Kingdom [99]. The list of bees in which ABPV was found increases constantly, including many *Bombus* species as well as a wide range of other bee species [54,100,101]. In non-bee Hymenoptera, ABPV was detected in *Ancistrocerus auctus, Polistes* spp., *V. germanica, Scolia flavifrons* and *Linepithema humile* [49,54,94].

#### 3.1.4. Israeli Acute Paralysis Virus (IAPV)

Israeli acute paralysis virus (IAPV) is a non-enveloped ssRNA (+) virus, belonging to *Apavirus* genus within the *Dicistroviridae* family, whose genome shows high homology to ABPV and KBV [88,102,103]. The virus has been isolated in Israel, but there are several known strains [102]. In honey bees, it induces disorientation, shivering wings, crawling, progressive paralysis and death within or ourside the nest [104].

The IAPV is widespread [102] and spillover cases were studied in a wide range of non-*Apis* bee species (Supplementary Table S1). Furthermore, the virus was found in the wasps, *V. germanica* and *V. vulgaris* [38,94] and in the ants, *Camponatus* spp. and *L. humile* [39,49]. Outside Hymenoptera, earwigs, spiders, moths, small hive beetles [39], and *V. velutina* [48] showed to act as IAPV incidental hosts.

#### 3.1.5. Slow Bee Paralysis Virus (SBPV)

Slow bee paralysis virus (SBPV) is an icosahedral non-enveloped ssRNA(+) virus from the *Iflavirus* genus within the *Iflaviridae* family [105,106]. The infection is responsible for paralysis of the first and second pairs of legs in roughly 12-day old honey bees and their sudden death [107,108].

Recently, it was found in wild *Bombus* spp., *E. nigriscens* and *O. bicornis* in Kyrgyzstan, Germany and Georgia [109], in the United Kingdom [110,111] and Belgium [112]. Furthermore, *E. nigriscens* and *O. bicornis* species in Kyrgyzstan, Germany and Georgia scored positive for SBPV infection [109] (Supplementary Table S1). No further spillover events have been reported so far in other arthropods.

#### 3.1.6. Chronic Bee Paralysis Virus (CBPV)

Chronic bee paralysis virus (CBPV) is an unclassified enveloped ssRNA (+) virus carachterized by articulate genome and association to a satellite virus (CBPSV) [113,114]. The infection causes a multifaceted disease encompassing different combinations of symptoms evidencing neurotropism like ataxia, incapability to fly, and trembling, as well as hairlessness and dark colour in the infected bees [114,115]. 

CBPV is capable to infect other insects. Spillover was reported in the wild in individuals of *B. dalhbomii* [92], *B. impatiens and B. ruderatus* [116], *B. pauloensis* [117], *B. terrestris* [92,116], *X. augusti and X. nigrocinta* [117], *X. dissimilis* [109], *H. amplilobus* [117] and *H. parallelum* [109]. Replicative CBPV was found in two ant species, *C. vagus and F. rufa* also [118].

#### 3.1.7. Sacbrood Virus (SBV)

SBV is a non-enveloped ssRNA (+) virus belonging to *Iflavirus* genus and *Dicistroviridae* family [27,28,119]. It is very common in honey bees, that exhibit symptoms in pre-imaginal stages coming into contact with the virus during the brood tending [27]. The virus is spread worldwide and genetic variants were identified in Korea (K-SBV), China (C-SBV), Thailand (T-SBV), Europe (E-SBV) and New Guinea (G-SBV) [120,121,122,123,124]. SBV was detected in a wide range of non-*Apis* bees (Supplementary Table S1) and other hymenopteran species (Supplementary Table S2) [38,39,54,110,116,125,126,127,128]. CBPV was detected in hoverflies, small hive beetles, spiders and lepidopterans also [39,82,129,130].

#### 3.1.8. Black Queen Cell Virus (BQCV)

BQCV belongs to the *Cripavirus* genus within *Dicistroviridae* family. As the other *Dicistroviridae*, BQCV is non-enveloped and ssRNA (+) virus [22,131,132]. 

Despite a high prevalence in adult honey bees [28,97,133,134,135], symptomatic infections occur in queen pupae and/or pre-pupae, that decompose in irregular, black cells [133,136].

BQCV is spread worldwideand affects several honey bee species and subspecies, like *A. mellifera*, *A. cerana indica*, *A. cerana japonica*, *A. dorsata* and *A. florea* [137]. The range of possible hosts is very wide and includes several wild hymenopteran species [37,38,39,46,54,109,110,125,126,138,139]. Small hive beetles, hoverflies, roaches, spiders and wax moths scored positive to BQCV also [39,129,130].

#### 3.1.9. Lake Sinai Virus (LSV)

LSV is an ssRNA(+) belonging to the *Sinhaliviridae* family and *Sinaivirus* genus, of which two strains have been identified so far: LSV-1 and LSV-2 [140]. The virus was discovered in honey bees sampled during a colony transhumance near the Lake Sinai, South Dakota, USA. LSV was reported as involved in the colony collapse disorder, despite both pathogenicity and epidemiology have not been clarified yet [70,141]. 

Cases of LSV spillover have been reported in *Andrena* spp. [37,127], *Bombus* spp., [85,112,127], and species belonging to the families of *Halictidae* and *Megachilidae* [127]. LSV has never been detected outside the *Apoidea* superfamily so far.

#### 3.1.10. *Apis mellifera* Filamentous Virus (*Am*FV)

*Am*FV is an unclassified dsDNA isolated from honey bees, whose relationship with the host and epidemiology are poorly studied. Originally, the pathogen was described as a rickettsia disease, but recently it has been recognized as a virus [142,143]. Severe infections of adult honey bees are associated to milk white hemolymph as a consequence of the high virion concentration. The infected bees show signs of weakness and tend to gather at the hive entrance. Nevertheless, the virus is weakly pathogenic and has low impact on bee lifespan [143,144,145,146]. 

Few spillover cases have been reported so far. They involved as alternative hosts *Andrena* spp. [37], *Bombus* spp. [147], *Osmia* spp. [37] and in *A. tumida* [148] (Supplementary Table S1).

#### 3.1.11. *Varroa destructor* Macula-like Virus (*Vd*MLV)

*Vd*MLV is an unclassified ssRNA(+) virus of the *Tymoviridae* family. The mite *V. destructor* is its primary host and the virus was found in the honey bees as a likely result of the trophic activity of the parasite [149]. Little knowledge is available for this virus. Few spillover cases have been reported so far about *Vd*MLV (Supplementary Table S1), all of them in the wild. Those involved *B. lapidarius, B. pascuorum* and *B. pratorum* as host species [112].

#### 3.1.12. Moku Virus

Moku virus is an unclassified ssRNA (+) *Iflavirus.* The virus was first discovered in *Vespula pensylvanica* in Hawaii, but it spread in honey bees too, often associated to *V. destructor* [150]. Since its discovery, Moku virus findings increased rapidly until the detection in a wide range of Hymenoptera species (Supplementary Table S1), that includes *Polistes* spp. [91], *Vespula* spp. [91,95,130,150], *V. velutina* [151] and *L. humile* [91]. Besides, Moku virus was found capable to infect the spiders *H. minitabunda* and *S. capensis* [91] (Supplementary Table S2).

### 3.2. Fungi

#### 3.2.1. *Nosema ceranae*

*Nosema ceranae* is a microsporidium that causes nosemosis type C in western honey bees [152,153]. It is an intracellular obligate parasite, infecting the ventricular epithelial cells [154,155]. The effects of *N. ceranae* infections can be recognized both at individual and colony levels, impacting the bee lifespan, inducing lethargic behaviour, reducing the pollen and honey harvest, and causing colony dwindling [156,157,158,159].

The main known spillover event occurred when the pathogen jumped from the Asian honey bee *A. ceranae*, which is deemed as the original host, to the western honey bee *A. mellifera* [152,153].

In addition to *A. cerana* and *A. mellifera*, the microsporidium was reported in several other Hymenoptera (Supplementary Table S1), including *A. ventralis*, *H. truncorum* and *Osmia* spp. [37], commercial and wild *Bombus* species [36,37,83,125,147,160,161,162], stingless bees, and *Polybya* spp. [163]. Besides, it was detected in the small hive beetle as well as in *A. tumida* [148,164]. Finally, the microsporidium was found in the regurgitated pellets of the European bee-eater *Merops apiaster* [165].

#### 3.2.2. *Nosema apis*

*Nosema apis* is the classic microsporidium infecting *A. mellifera*, which is responsible for the nosemosis Type A [166]. Like the other microsporidians, it is an intracellular obligate parasite. It causes, in contrast to *N. ceranae*, severe dysentery that impacts mainly the colony foragers [166,167,168]. Presently, its spread is limited to specific ecological niches as a possible consequence of the competition with the predominant *N. ceranae* [71,157]. *N. apis* was detected in commercial *B. terrestris* colonies [20], but the transmission route remained unclarified.

#### 3.2.3. *Ascosphaera apis*

The fungus *Ascosphaera apis* is a honey bee pathogen responsible for the mycosis called chalkbrood disease [169,170]. The infection occurs by spore ingestion in bee larvae, especially in those of the fifth instar, that reduces food consumption and prevents eating [169,170]. The proliferating mycelium invades the larval body, which is transformed into a chalk-like “mummy”, so the disease name [169,171,172].

Despite the disease is typical to the honey bees, artificial infections showed the pathogen capability to colonize the intestine of *B. terrestris* adults and larvae [20].

### 3.3. Bacteria

#### 3.3.1. *Melissococcus plutonius*

The bacterium *M. plutonius* is the Gram-negative coccus representing the etiological agent of the European foulbrood disease [173,174].

The pathogen is spread worldwide and infects the brood, which dies by undernutrition [175,176]. The infected larvae become flaccid and yellowish by 5 days after infection [173,175,176].

In the United Kingdom, *M. plutonius* was found to impair the development of *B. terrestris* colonies [20].

#### 3.3.2. *Spiroplasma apis*

*Spiroplasma apis* is a small, helical and motile Gram-positive *Eubacterium* deprived of a cell wall [177,178]. The bacterium was isolated in France from colonies showing symptoms of “May disease” [179]. *S. apis* is lethal to the honey bees when ingested, and the infection may spread by faecal contamination [179].

Strains of *S. apis* were isolated and detected in wild specimens belonging to *B. atratus* [125] and *O. bicornis* [37], with unknown effects.

#### 3.3.3. *Spiroplasma melliferum*

*Spiroplasma melliferum* is another Eubacterium isolated from the honey bees [180]. The *S. melliferum* infection has similar symptoms and transmission route as *S. apis*, although less virulent [179,180]. As for *S. apis, S. melliferum* spillover was observed occasionally (Supplementary Table S1). This is the case of *O. bicornis* individuals, that were found infected in Belgium [37].

#### 3.3.4. *Wolbachia* spp.

*Wolbachia* spp. are Gram-negative intracellular bacterial symbionts, which can infect the cells of both female honey bees and drones [181,182]. *Wolbachia* spp. impacts the host reproduction. The vertical transmission via the eggs represents the main transmission route to persist in honey bee populations [183,184].

During a national survey in the U.S.A. (Supplementary Tables S1 and S2), several arthropods scored positive to *Wolbachia* spp.: *Andrena* spp., several *Bombus* species, *Lasioglossum* spp., *Halictus* spp., *D. sylvestris*, *V. germanica* and *V. vulgaris*, and two hoverflies [128].

### 3.4. Trypanosomatidae

#### 3.4.1. *Lotmaria passim*

*Lotmaria passim* is a trypanosomatid with a single flagellum, capable to colonize the digestive tract of *A. mellifera* [185,186]. The parasite spreads within the colony by fecal contact, and the transmission occur via the oro-faecal route [187,188]. The infection impacts the colony by altering behaviour and lifespan of the infected bees [141,189]. *L. passim* is spread worldwide. The colonization implied the replacing of the other honey bee trypanosomatids *Crithidia mellificae* [190,191].

Besides, *L. passim* is present in bumblebee species (Supplementary Table S1) namely th South American *B. funebris, B. dalhbomii, B. opifex, B. ruderatus* and *B. terrestris* [92,147].

*L. passim* was found also in the small hive beetle, *A. tumida*, as a possible result of the feeding behaviour of this scavenger [81,148].

#### 3.4.2. *Crithidia mellificae*

*Crithidia mellificae* is another trypanosomatid which can replicate in the honey bee intestine to survive [185,186]. Transmission route and impact on bees are very similar to the other parasite *L. passim* [187,188,192]. *C. mellificae* was almost completely replaced by *L. passim* and its infection has been rarely observed [185,193,194].

Despite that, one spillover case was observed in *A. tumida*, that live in contact with bee colony debris [81].

#### 3.4.3. *Crithidia bombi*

*Crithidia bombi* is a trypanosomatid infecting *B. terrestris* colonies [195,196]. The infection occurs during the external activity of the forager bumblebees [197,198] and, back to the nest, it spreads by fecal contamination to the other workers [199,200]. *C. bombi* may harm the bumblebee populations as hibernating queens may reduce the success in founding the colonies and remarkably lower their fitness [201]. On queen emergence from the diapause, *C. bombi* infections grow together with the colony that is being established [200].

*C. bombi* is transmitted during the foraging activity. The pathogen was detected in the wild on *A. vaga* and *O. bicornis* individuals [37] and in small hive beetles collected from the nest of honey bee colonies [148]. Artificial infections showed that *C. bombi* can replicate in *O. lignaria*, *M. rotundata* and *H. ligatus* [202,203].

### 3.5. Neogregarine

#### Apicystis bombi

*Apicystis bombi* is a parasite found primarily in bumblebees. It was found to occur also in honey bees from Europe and North America [204,205,206]. Upon the ingestion of the oocytes by the bee, the sporozoites develop and migrate to the fat body, where they develop, multiply and disrupt the adipose tissue. The infection increases the worker mortality rate and, due to the fat body disruption, both queen survival to hibernation and colony foundation success are impaired [84,207,208].

Likely, the infection occurs via contact on contaminated flowers [208]. Indeed, *A. bombi* was found in wild species also, namely *A. vaga*, *A. ventralis*, *H. truncorum*, *O. bicornis* and *O. cornuta* [37].

## 4. Materials and Methods

### Protocol and Literature Search

This systematic review was carried out according to the Preferred Reporting Items for Systematic Review and Meta-Analysis (PRISMA) protocols [209]. The research question to be reviewed was: “Which honey bee pathogens may generate spillover to managed and wild Hymenoptera species and, more in general, to the arthropofauna?”

The search intentionally excluded arthropods living in close contact with the honey bees that, like *V. destructor*, are obligate parasites.

The article search was carried out in PubMed, Web of Science, Science Direct, Google Scholar, and Scopus scientific databases for studies aimed to assess the detection of spillover cases of honey bee pathogens. Filters were used to select articles published from January 1960 to April 2021. The last search date was 31 May 2021.

The following search strategy was designed and utilized: “Honey Bee Pathogens” OR “Bumblebee Pathogens” OR “Spillover” OR “Spill-over ” OR “Inter species transmission” OR “Inter taxa transmission” OR “Host species transmission” OR “*Apis mellifera*” OR “Honey Bee Diseases” OR “Honey Bee Virus” OR “Honey Bee Bacteria” OR “Honey Bee Microsporidia” OR “Honey Bee Protozoa” OR “Managed Bees” OR “Wild Bees” OR “Commercial Bees” OR “Artificial Infection” OR “Replicative Virus” OR “Bumblebees” OR “Colony Collapse Disorder” OR “Deformed Wing Virus” OR “Acute Bee Paralysis Virus” OR “Israeli Acute Paralysis Virus” OR “Black Queen Cell Virus” OR “Sacbrood Virus” OR “*Apis mellifera* Filamentous Virus” OR “Kashmir Bee Virus” OR “Slow Bee Paralysis Virus” OR “Lake Sinai Virus” OR “*Varroa destructor*” OR “Macula-like Virus” OR “*Nosema apis*” OR “*Nosema ceranae*” OR “*Nosema bombi*” OR “*Spiroplasma*” OR “*Ascosphaera*” OR “*Apicystis*” OR “Arthopods” OR “Entomofauna” OR “Hive Hosts” OR “Hive” OR “Free-Ranging Insect” OR “Bee Interaction” OR “*Varroa destructor*”. The logical operator “OR” was used to combine the descriptors.

Studies carried out both in field and laboratory conditions were selected. Besides, studies that did not assess whether the presence of the honey bee pathogens could be related to external contamination were not included. The detected active replication of honey bee viruses was also reported in the Supplementary Materials with an asterisk.

Duplicate studies were excluded. The search and screening for titles, abstracts and results were carried out independently by the authors, including all articles, letters, notes, scientific notes and communications aimed to assess a spillover case of honey bee pathogens and excluding reviews, books, book chapters and theses.

The potentially eligible research articles were read and reviewed independently by the authors and the data were compared to ensure integrity and reliability.

For each article included in this review, relevant information related to the authors, publication year, host species, host conditions, host stage, pathogens and prevalence were extracted. The data from the eligible studies are expressed in the Supplementary Materials and Figures. The authors provided a narrative synthesis of the results for each pathogen capable to generate a spillover case, according to the main characteristics and results related to the topic addressed.

## 5. Conclusions

This review shows that, in recent years, the frequency of recorded spillover cases of honey bee pathogens to other arthropods, including wild bees, has dramatically increased. Certainly, human movements and globalization have fostered the inflow of novel pathogenic microorganisms, often with detrimental consequences. However, it should also be considered that the analytical methods currently available give impulse to the research on bee pathology, increasing the chance to identify interspecific transmission events.

The host plasticity shown by some honey bee pathogens raises ecological concern for the potential negative consequences on the pollinating entomofauna and ecosystems in general. Despite the fact that research on these pathogens has significantly improved, we have limited knowledge of their potential impact on other bees, insects, and arthropods in general and the cascade of environmental effects. Laboratory studies are not sufficient to cover this gap, for the intricate interaction of the involved biotic and abiotic factors. For the same reasons, the exploitation of these pathogens in the control of arthropods considered as pests (e.g., *A. tumida*, *G. melonella*, *V. velutina*, *L. humile*) should be considered with extreme carefulness.

The tight interaction between honey bees and the other environmental components suggests a holistic approach to the study of bee diseases, including their control. Indeed, pathogens may survive in alternate hosts, generating spillback events and possibly jeopardizing the efficacy of the treatments. This emphasizes the beekeeper’s responsibility to maintain healthy colonies to benefit both their production and the environment.

Spillover of honey bee pathogens may have undetected yet important repercussions on the health and functioning of an ecosystem. Health management of honey bee colonies is of high importance in this context. Honey bees and the beekeeping industry should, therefore, undertake an essential role in the One Health concept. This requires the adoption of dedicated research actions.

## Data Availability

MDPI Research Data Policies.

## Supplementary Materials

The following are available online at https://www.mdpi.com/article/10.3390/pathogens10081044/s1, Table S1: Bee pathogen spillover and prevalence identified in hymenopteran hosts, of which are reported condition, stage, geographical area and year, Table S2: Bee pathogen spillover and prevalence identified in arthropod hosts, of which are reported condition, stage, geographical are and year [210,211,212,213,214,215,216,217,218,219,220,221,222,223,224,225,226,227,228,229,230,231,232].
