# Supplementary material for: Pathogens Spillover from Honey Bees to Other Arthropods
Source: Pathogens. 2021 Aug 17;10(8):1044. doi: 10.3390/pathogens10081044 (PMC8400633; doi:10.3390/pathogens10081044)
Supplement: Supplementary file 1 [file pathogens-10-01044-s001.zip › pathogens-1314272-supplementary.pdf]

Table S1. Bee pathogen spillover and prevalence identified in hymenopteran hosts, of which are reported condition, stage, geographical area and year.

Host			Geographic area	Year	Pathogen	Prevalence (%)	Reference
Taxon	Condition	Stage					
<u>**BEE**</u>							
<u>Andrenidae</u>							
Andrena spp.	Wild	Adult	France	2021	ABPV	25.0%	[100]
					IAPV	25.0%	
					BQCV	25.0%	
			U.S.A.	2005	BQCV[A]	25.0%	[38]
					SBV[A]	12.5%	
					DWV[A]	62.5%	
				2008	*Wolbachia* spp.	69.2%	[128]
					Ascosphaera spp.	84.6%	
Andrena bicolor	Wild	Adult	Germany	2014	DWV	-	[109]
			Georgia		DWV	-	
Andrena haemorrhoa	Wild	Adult	Germany	2014	DWV*	-	[109]
Andrena helianthi	Wild	Adult	U.S.A.	2013	IAPV	16.7%	[127]
					SBV	41.7%	
					DWV	66.7%	
					LSV	25.0%	
Andrena helianthiformis	Wild	Adult	U.S.A.	2013	IAPV	16.7%	[127]
					SBV	41.7%	
					DWV	66.7%	
					LSV	25.0%	
Andrena helvola	Wild	Adult	Germany	2014	DWV	-	[109]
Andrena labialis	Wild	Adult	France	2021	ABPV	100%	[100]
Andrena rudbeckiae	Wild	Adult	U.S.A.	2013	IAPV	16.7%	[127]
					SBV	41.7%	
					DWV	66.7%	
					LSV	25.0%	
Andrena thoracica	Wild	Adult	Kyrgyzstan	2014	DWV	100%	[109]
Andrena trimmerana	Wild	Adult	Georgia	2014	DWV	-	[109]
Andrena ventralis	Wild	Adult	Belgium	2012	LSV[B]	100%	[37]
					*Am*FV[B]	100%	
					N. ceranae[B]	100%	
					Apicystis bombi[B]	100%	
Andrena vaga	Wild	Adult	Belgium	2012	BQCV[C]	33.4%	[37]
					SBV[C]	33.4%	
					LSV[C]	100%	
					*Am*FV[C]	100%	
					C. bombi[C]	100%	
					Apicystis bombi[C]	100%	
Andrena wilkella	Wild	Adult	U.S.A.	2013	IAPV	16.7%	[127]
					SBV	41.7%	
					DWV	66.7%	
					LSV	25.0%	
Melandrena spp.	Wild	Adult	U.S.A.	2014	BQCV	31.1%	[210]
					SBV	6.70%	
					DWV	10.0%	
<u>Apidae</u>							
Anthophora plumipes	Wild	Adult	Georgia	2014	ABPV	-	[109]
			Germany		ABPV	-	
			Kyrgyzstan		ABPV	-	
			Georgia		IAPV	-	
			Germany		IAPV	-	
			Kyrgyzstan		IAPV	-	
			Georgia		BQCV	-	
			Germany		BQCV	-	
			Kyrgyzstan		BQCV	-	
			Georgia		SBV	-	
			Germany		SBV	-	
			Kyrgyzstan		SBV	-	
			Georgia		DWV	-	
			Germany		DWV	-	
			Kyrgyzstan		DWV	-	
			Georgia		KBV	-	
			Germany		KBV	-	
			Kyrgyzstan		KBV	-	

Anthophora plumipes	Wild	Adult	Georgia	2014	SBPV	-	[109]
			Germany		SBPV	-	
			Kyrgyzstan		SBPV	-	
Bombus spp.	Wild	Adult	France	2021	ABPV	58.6%	[100]
					IAPV	27.6%	
					BQCV	14.4%	
			U.S.A.	2005	BQCV[D]	33.3%	[38]
			France	2021	SBV	82.8%	[100]
			U.S.A.	2005	SBV[D]	100%	[38]
			France	2021	DWV	6.89%	[100]
			U.S.A.	2005	DWV[D]	100%	[38]
					KBV[D]	100%	[38]
			New Zealand	2019	KBV	-	[91]
			United Kingdom	2013	*N. ceranae*	21.0%	[162]
Bombuis agrorum	Wild	Adult	United Kingdom	1964	ABPV*	-	[99]
Bombus atratus	Wild	Adult	Colombia	2013	ABPV[E]	11.0%	[125]
			Argentina	2011	BQCV[F]	74.0%	
			Colombia	2013	BQCV[E]	74.0%	[126]
					SBV[E]	16.0%	[125]
			Argentina	2011	SBV[F]	100%	[126]
			Colombia	2013	DWV[E]	100%	[125]
			Argentina	2011	DWV[F]	100%	[126]
			Colombia	2013	LSV	68.0%	[125]
			Colombia	2013	*N. ceranae*[E]	100%	
			Argentina	2009	*N. ceranae*	12.5%	[160]
			Colombia	2013	*Spiroloplasma apis*	5.00%	[125]
Bombus auricomus	Wild	Adult	U.S.A.	2013	IAPV	3.13%	[127]
					BQCV	15.6%	
					SBV	6.25%	
					DWV	62.5%	
					LSV	3.13%	
Bombus bimaculatus	Wild	Adult	U.S.A.	2015	BQCV*	65.9%	[139]
					DWV*	9.30%	
Bombus bellicosus	Wild	Adult	Argentina	2009	*N. ceranae*	6.25%	[160]
Bombus brasiliensis	Wild	Adult	Argentina	2015	*N. ceranae*	100%	[161]
Bombus cryptarum	Wild	Adult	Kyrgyzstan	2014	SBPV	-	[109]
Bombus dahlbomii	Wild	Adult	Chile	2017	ABPV	7.69%	[92]
					BQCV	23.08%	[92]
					KBV	3.08%	
					CBPV	6.15%	
					L. passim	6.15%	
Bombus funebris	Wild	Adult	Peru	2018	*Am*FV	100%	[147]
			Bolivia		*L. passim*	40.0%	
Bombus griseocollis	Wild	Adult	U.S.A.	2013	IAPV	12.1%	[127]
					BQCV	6.10%	
					SBV	51.5%	
					DWV	45.5%	
					LSV	15.1%	
Bombus hortorum	Wild	Adult	United Kingdom	1964	ABPV*	-	[99]
				2014	ABPV	2.50%	[110]
			Slovenia	2018	ABPV	66.0%	[213]
			United Kingdom	2014	BQCV	11.0%	[110]
			Slovenia	2017	BQCV	100%	[213]
			United Kingdom	2014	SBPV	35.0%	[110]
			Slovenia	2018	SBV	33.0%	[213]
			Slovenia	2018	LSV	33.0%	[213]
				2008	Microsporida	42.9%	[128]
	Artificial Infection		United Kingdom	2014	*N. ceranae*	25.0%	[83]
	Wild		U.S.A.	2008	*Wolbachia* spp.	85.7%	[128]
					Ascosphaera spp.	85.7%	
Bombus humilis	Wild	Adult	France	2021	ABPV[G]	100%	[100]
					SBV[G]	100%	
			Slovenia	2018	BQCV	100%	[213]
					DWV	50.0%	
Bombus huntii	Wild	Adult	U.S.A.	2011	BQCV*	-	[211]
	Laboratory				BQCV*	-	
	Wild				DWV*	-	[212]
	Laboratory				DWV*	-	

Bombus ignitus	Commercial	Adult	Korea	2010	IAPV	-	[116]
					BQCV	-	
					SBV	-	
					DWV	-	
					KBV	-	
					CBPV	-	
Bombus impatiens	Commercial	Adult	Mexico	2014	ABPV	2.00%	[23]
					IAPV	2.00%	
	Wild		U.S.A.	2011	IAPV	31.%	[93]
					BQCV[H]	60.0%	[38]
					BQCV	69.0%	[93]
					SBV	23.0%	
	Commercial		Mexico	2014	DWV	2.00%	[23]
	Wild		U.S.A.	2005	DWV[H]	100%	[38]
					DWV	62.0%	[93]
	Commercial		Mexico	2014	KBV	2.00%	[23]
					CBPV	3.34%	
Bombus laesus	Wild	Adult	Kyrgyzstan	2014	BQCV*	-	[109]
Bombus lapidarius	Wild	Adult	United Kingdom	2014	ABPV	22.0%	[110]
			Slovenia	2017	ABPV	30.0%	[213]
			United Kingdom	2014	BQCV	7.50%	[110]
			Slovenia	2017	BQCV	60.0%	[213]
	Artificial Infection		Slovenia	2018	BQCV	90.0%	[213]
	Wild		United Kingdom	2014	DWV*[I]	9.14%	[83]
					DWV	2.00%	[110]
					DWV	25.0%	[128]
			Slovenia	2017	DWV	10.0%	[213]
			Belgium	2013	SBPV	3.57%	[112]
			United Kingdom	2014	SBPV	1.00%	[110]
			Germany		SBPV	-	[109]
			Kyrgyzstan		SBPV	-	
			Belgium	2013	LSV	3.57%	[112]
			Slovenia	2018	LSV	9.11%	[213]
			Belgium	2013	*Vd*MLV	3.57%	[112]
			U.S.A.	2008	Microsporidia	50.0%	[128]
	Artificial Infection		United Kingdom	2014	*N. ceranae*[I]	9.14%	[83]
	Wild		U.S.A.	2008	*Wolbachia* spp.	75.0%	[128]
					Ascosphaera spp.	100%	
Bombus lucorum	Wild	Adult	United Kingdom	1964	ABPV*	-	[99]
				2014	ABPV	3.00%	[110]
					BQCV	2.00%	
	Artificial Infection				DWV*	30.0%	[83]
	Wild				DWV	3.00%	[110]
			Georgia		DWV	-	[109]
			Kyrgyzstan		DWV	-	
			United Kingdom		SBPV	3.00%	[110]
			Georgia		SBPV	-	[109]
			Kyrgyzstan		SBPV	-	
Bombus monticola	Artificial Infection	Adult	United Kingdom	2014	*DWV**	100%	[83]
Bombus morio	Wild	Adult	Argentina	2009	*N. ceranae*	12.5%	[160]
Bombus opifex	Wild	Adult	Peru	2018	*Am*FV	100%	[147]
			Bolivia		*N. ceranae*	28.6%	
			Bolivia		*L. passim*	16.7%	
Bombus pascuorum	Wild	Adult	France	2021	ABPV	71.4%	[100]
			United Kingdom	1964	ABPV*	-	[99]
				2014	ABPV	1.50%	[110]
			Slovenia	2017	ABPV	7.69%	[213]
			Slovenia	2018	ABPV	3.40%	
			France	2021	IAPV	14.3%	[100]
			Spain	2016	BQCV	9.40%	[214]
			France	2021	BQCV	14.3%	[100]
			United Kingdom	2014	BQCV	5.00%	[110]
			Slovenia	2017	BQCV	23.1%	[213]
			Slovenia	2018	BQCV	65.5%	
			France	2021	SBV	100%	[100]
			Slovenia	2017	SBV	30.8%	[213]
			Slovenia	2018	SBV	48.0%	
			Spain	2016	DWV	6.30%	[214]
			Germany	2004	DWV	100%	[69]

Bombus pascuorum	Artificial Infection	Adult	United Kingdom	2014	DWV*	6.67%	[83]
	Wild		U.S.A.	2008	DWV	5.56%	[128]
			United Kingdom	2016	DWV	30.7%	[36]
			Belgium	2013	SBPV	32.5%	[112]
			United Kingdom	2014	SBPV	1.50%	[110]
			Germany	2014	SBPV	-	[109]
			Belgium	2013	LSV	5.00%	[112]
			Slovenia	2017	LSV	11.5%	[213]
			Slovenia	2018	LSV	24.1%	
					*Vd*MLV	16.3%	
			U.S.A.	2008	Microsporidia	38.9%	[128]
			United Kingdom	2016	*N. ceranae*	12.3%	[36]
			U.S.A.	2008	*Wolbachia* spp.	75.0%	[128]
					Ascosphaera spp.	22.2%	
Bombus pauloensis	Wild	Adult	Bolivia	2018	*Am*FV	100%	[147]
			Argentina		CBPV	100%	[117]
			Bolivia	2018	*N. ceranae*	33.4%	[147]
			Uruguay	2014	DWV	39.0%	[215]
					BQCV	80.9%	
					ABPV	20.0%	
					SBV	27.0%	
Bombus pratorum	Wild	Adult	Belgium	2013	*Vd*MLV	16.3%	[112]
				2008	Microsporidia	14.3%	[128]
					Wolbachia spp.	71.4%	
					Ascosphaera spp.	100%	
Bombus ruderatus	Wild	Adult	Chile	2017	ABPV	28.97%	[92]
			France	2021	ABPV	50.0%	[100]
			Chile	2017	BQCV	22.07%	[92]
			France	2021	BQCV	50.0%	[100]
					SBV	100%	
			Chile	2017	DWV	5.52%	[92]
			France	2021	DWV	25%	[100]
			Chile	2017	KBV	2.07%	[92]
			Chile	2017	CBPV	2.76%	[92]
					L. passim	2.76%	
Bombuis ruderaius	Wild	Adult	United Kingdom	1964	ABPV*	-	[99]
Bombus rupestris	Wild	Adult	Germany	2014	BQCV	-	[109]
Bombus soroeensis	Wild	Adult	Kyrgyzstan	2014	BQCV*	-	[109]
Bombus sylvarum	Wild	Adult	Georgia	2014	DWV	-	[109]
			Slovenia	2018	BQCV	91.7%	[213]
					SBV	16.7%	
					LSV	8.30%	
Bombus ternarius	Wild	Adult	U.S.A.	2005	IAPV[J]	100%	[38]
					BQCV	50.0%	
					SBV[J]	50.0%	
					DWV[J]	100%	
Bombus terrestris	Wild	Adult	Chile	2017	ABPV	5.56%	[92]
			France	2021	ABPV	72.1%	[100]
			United Kingdom	2014	ABPV	10.0%	[110]
				1964	ABPV*	-	[99]
				2014	ABPV	6.00%	[110]
	Artificial Infection		Belgium	2014	IAPV	60.0%	[216]
				2015	IAPV	-	[217]
					IAPV	-	[218]
				2018	IAPV	-	[219]
	Wild		France	2021	IAPV	16.3%	[100]
			Korea	2014	BQCV	7.09%	[138]
			Chile	2017	BQCV	33.3%	[92]
			France	2021	BQCV	20.9%	[100]
			United Kingdom	2014	BQCV	5.00%	[110]
			Germany		BQCV*	-	[109]
			Georgia		BQCV*	-	
	Commercial	Adult	Kyrgyzstan	2014	BQCV*	-	
	Wild		France	2021	SBV	95.3%	[100]
			United Kingdom	2014	SBV	2.50%	[110]
			Korea	2010	SBV	-	[116]
			Korea	2014	DWV	7.09%	[138]
			Chile	2017	DWV	13.1%	[92]
			France	2021	DWV	18.6%	[100]

Bombus terrestris	Wild	Adult	U.S.A.	2008	DWV	29.3%	[128]
			United Kingdom	2014	DWV	6.00%	[110]
			United Kingdom	2016	DWV	22.3%	[36]
			Germany	2014	DWV*	-	[109]
			Georgia	2014	DWV*	-	
	Artificial Infection		Kyrgyzstan	2014	DWV*	-	[109]
			United Kingdom	2014	DWV*	5.30%	[83]
				2015	DWV	50.0%	[84]
	Commercial		Germany	2004	DWV*	100%	[69]
			Korea	2010	DWV	-	[116]
			United Kingdom	2013	DWV	15.0%	[20]
					DWV	15.0%	
			Italy	2021	DWV*	100%	[68]
		Pupae	Italy	2021	DWV*	100%	
		Larvae			DWV*	100%	
		Eggs			DWV*	100%	
	Artificial Infection	Adult	U.S.A.	2020	DWV*	-	[55]
		Larvae			DWV*	-	
	Artificial Infection	Adult	Belgium	2014	KBV	100%	[216]
	Wild		Chile	2017	KBV	6.06%	[92]
					CBPV	1.52%	[92]
	Commercial		Korea	2010	CBPV	-	[116]
	Artificial Infection		Belgium	2015	SBPV	-	[218]
			United Kingdom	2017	SBPV	100%	[111]
	Wild		United Kingdom	2014	SBPV	6.00%	[110]
			U.S.A.	2008	Microsporidia	8.62%	[128]
	Artificial Infection		United Kingdom	2014	*N. ceranae*	1.18%	[83]
				2016	*N. ceranae*	-	[162]
	Wild		United Kingdom	2016	*N. ceranae*	38.7%	[36]
	Commercial		United Kingdom	2015	*N. ceranae*	20.0%	[23]
	Commercial		United Kingdom	2013	*N. apis*	15.0%	[20]
	Wild		Chile	2017	*L. passim*	4.04%	[92]
	Commercial		United Kingdom	2013	*M. plutonius*	15.0%	[20]
	Wild		U.S.A.	2008	*Wolbachia* spp.	91.4%	[128]
					Ascosphaera spp.	15.5%	
	Artificial Infection	Larvae	Belgium	2016	*Ascophaera apis*	-	[220]
		Adult			*Ascophaera apis*	-	
Bombus vagans	Wild	Adult	U.S.A.	2005	IAPV[K]	100%	[20]
				2005	BQCV[K]	100%	
				2011	BQCV	100%	[93]
				2015	BQCV*	86.3%	[139]
				2005	SBV[K]	100%	[38]
				2011	SBV	50.0%	[93]
				2005	DWV[K]	100%	[38]
				2011	DWV	50.0%	[93]
				2015	DWV*	9.20%	[139]
Bombus vestalis	Wild	Adult	Germany	2014	BQCV*	-	[109]
Bombus ephippiatus	Wild	Adult	Mexico	2020	DWV	100%	[221]
					BQCV	100%	
Bombus steindachner	Wild	Adult	Mexico	2020	DWV	100%	[221]
Ceratina dupla	Wild	Adult	U.S.A.	2005	DWV	66.4%	[38]
Ceratina smaragdula	Wild	Adult	Hawaii	2015	DWV	27.0%	[222]
Eucera spp.	Wild	Adult	France	2021	ABPV	22.%	[100]
					IAPV	77.8%	
					BQCV	66.7%	
					SBV	55.6%	
					DWV	11.1%	
Eucera nigrescens	Wild	Adult	Georgia	2014	ABPV[L]	100%	[109]
					IAPV[L]	100%	
					BQCV[L]	100%	
					SBV[L]	100%	
					DWV[L]	100%	
					KBV[L]	100%	
					SBPV[L]	100%	
Melecta albifrons	Wild	Adult	Germany	2014	SBPV	-	[109]
			Georgia		SBPV	-	
Melipona fasciculata	Wild	Adult	Brasil	2015	*N. ceranae*	100%	[163]
Melipona mandacaia	Wild	Adult	Brasil	2015	*N. ceranae*	100%	[163]
Melipona marginate	Wild	Adult	Brasil	2015	*N. ceranae*	100%	[163]

Melipona quadrifasciata anthidiodes	Wild	Adult	Brasil	2015	*N. ceranae*	100%	[163]
Melipona rufiventris	Wild	Adult	Brasil	2015	*N. ceranae*	100%	[163]
Melipona scutellaris	Wild	Adult	Brasil	2015	ABPV	100%	[223]
Melipona colimana	Wild	Adult	Mexico	2019	DWV	33.3%	[221]
				2020	DWV	50.0%	[224]
				2019	BQCV	100%	[221]
				2020	BQCV	50.0%	[224]
Melissodes bimaculata	Wild	Adult	U.S.A.	2013	IAPV	20.0%	[127]
					SBV	80.0%	
					DWV	60.0%	
					LSV	10.0%	
Nomada distinguenda	Wild	Adult	France	2021	ABPV[M]	100%	[100]
					IAPV[M]	100%	
					BQCV[M]	50.0%	
					SBV[M]	50.0%	
Peponapis fervens	Wild	Adult	Argentina	2018	CBPV	100%	[117]
Plebeia emerinoides	Wild	Adult	Argentina	2017	IAPV	12.5%	[101]
Plebeia droryana	Wild	Adult	Argentina	2017	IAPV	20.0%	[101]
Scaptotrigona jujuyensis	Wild	Adult	Argentina	2015	*N. ceranae*	100%	[163]
Scaptotrigona mexicana	Wild	Adult	Mexico	2015	DWV	60.0%	[225]
				2020	DWV	60.0%	[221]
					BQCV	40.0%	
Tetragonisca fiebrigi	Wild	Adult	Argentina	2017	IAPV	3.70%	[101]
				2014	DWV	48.1%	[163]
					N. ceranae	100%	
				2015	*N. ceranae*	100%	
Trigona spinipes	Wild	Adult	Argentina	2017	IAPV	25.0%	[101]
Trigona fulviventris	Wild	Adult	Mexico	2020	DWV	100%	[221]
Nannotrigona perilapodes	Wild	Adult	Mexico	2020	DWV	100%	[221]
Tetragonula hockingsi	Artificial Infection	Adult	Australia	2019	*N. ceranae*	67.0%	[226]
Xylocopa atamisquensis	Wild	Adult	Argentina	2018	CBPV	50.0%	[117]
Xylocopa augusti	Laboratory	Larvae	Argentina	2013	DWV	30.0%	[85]
	Wild	Adult		2018	CBPV	87.5%	[117]
Xylocopa dissimilis	Wild	Adult	Kyrgyzstan	2014	CBPV	-	[109]
Xylocopa iris	Wild	Adult	France	2021	ABPV[N]	100%	[100]
					BQCV[N]	100%	
Xylocopa nigrocinta	Wild	Adult	Argentina	2018	CBPV	66.7%	[117]
Xylocopa violacea	Wild	Adult	France	2021	ABPV[O]	100%	[100]
					BQCV[O]	100%	
					SBV[O]	100%	
					DWV[O]	100%	
Xylocopa virginica	Wild	Adult	U.S.A.	2005	BQCV[P]	50.0%	[38]
				2011	BQCV	100%	[93]
					SBV	100%	
				2005	DWV[P]	75.0%	[38]
Colletidae							
Colletes spp.	Wild	Adult	Mexico	2020	DWV	100%	[221]
					BQCV	100%	[221]
Hylaeus dilatatus	Wild	Adult	France	2021	ABPV	20.0%	[100]
					IAPV	40.0%	
Halictidae							
Agapostemon viriscens	Wild	Adult	U.S.A.	2013	IAPV	55.3%	[127]
					SBV	41.7%	
					DWV	66.7%	
					LSV	5.26%	[127]
Augochlora pura	Wild	Adult	U.S.A.	2005	IAPV[Q]	100%	[38]
					DWV[Q]	100%	
				2013	BQCV	16.7%	[127]
Augochlorella auratus	Wild	Adult	U.S.A.	2013	SBV	100%	[127]
					LSV	33.3%	
Halictus spp.	Wild	Adult	France	2021	ABPV	75.0%	[100]
					IAPV	25.0%	
					BQCV	25.0%	
					SBV	25.0%	
					DWV	50.0%	
			U.S.A.	2011	DWV	25.0%	[93]
				2008	*Wolbachia spp.*	70.0%	[128]
					Ascosphaera spp.	70.0%	
Halictus fulvipes	Wild	Adult	France	2021	ABPV	58.8%	[100]

Halictus fulvipes	Wild	Adult	France	2021	BQCV	11.8%	[100]
					SBV	29.4%	
Halictus ligatus	Wild	Adult	U.S.A.	2013	IAPV	3.13%	[127]
					BQCV	15.6%	
					SBV	6.25%	
					DWV	62.5%	
					LSV	3.13%	
Halictus maculatus	Wild	Adult	France	2021	IAPV	100%	[100]
Halictus patellatus	Wild	Adult	France	2021	ABPV	100%	[100]
Halictus parallelum	Wild	Adult	U.S.A.	2013	IAPV	3.13%	[127]
					BQCV	15.6%	
					SBV	6.25%	
					DWV	62.5%	
			Georgia	2014	CBPV	-	[109]
			Kyrgyzstan		CBPV	-	
			U.S.A.	2013	LSV	3.13%	[127]
Halictus rubicundus	Wild	Adult	U.S.A.	2013	IAPV	3.13%	[127]
					BQCV	15.6%	
					SBV	6.25%	
					DWV	62.5%	
					LSV	3.13%	
Halictus simplex	Wild	Adult	France	2021	BQCV	100%	[100]
Halictus tectus	Wild	Adult	France	2021	BQCV	50.0%	[100]
					SBV	25.0%	[100]
Halictus ligatus	Artificial Infection	Adult	U.S.A.	2020	*C. bombi*	-	[203]
					C. mellificae	-	
Halictillus amplilobus	Wild	Adult	Argentina	2018	CBPV	100%	[117]
Lasioglossum spp.	Wild	Adult	U.S.A.	2008	Microsporidia	2.22%	[128]
					Wolbachia spp.	40.0%	
					Ascosphaera spp.	51.1%	
Lasioglossum crassepunctatum	Wild	Adult	France	2021	ABPV[R]	100%	[100]
					IAPV[R]	100%	
					BQCV[R]	100%	
					SBV[R]	100%	
Lasioglossum dialectus	Wild	Adult	U.S.A.	2013	IAPV	3.13%	[127]
					BQCV	15.6%	
					SBV	6.25%	
					DWV	62.5%	
					LSV	3.13%	
Lasioglossum malachurum	Wild	Adult	France	2021	ABPV	26.2%	[100]
					IAPV	33.8%	
					BQCV	53.8%	
					SBV	43.1%	
Lasioglossum nigripes	Wild	Adult	France	2021	BQCV	100%	[100]
Lasioglossum pauperatum	Wild	Adult	France	2021	IAPV	16.7%	[100]
					BQCV	33.4%	
					SBV	33.4%	
Lasioglossum pauxillum	Wild	Adult	France	2021	BQCV	100%	[100]
					SBV	33.4%	
					DWV	33.4%	
<u>Megachilidae</u>							
Anthidium manicatum	Wild	Adult	France	2021	ABPV[S]	100%	[100]
					IAPV[S]	100%	
Heriades truncorum	Wild	Adult	Belgium	2012	BQCV[T]	66.7%%	[37]
					N. ceranae	100%	
					Apicystis bombi[T]	33.3%	
Hoplitis adunca	Wild	Adult	France	2021	IAPV[U]	100%	[100]
					BQCV[U]	100%	
					SBV[U]	100%	
Megachile albisecta	Wild	Adult	France	2021	SBV	100%	[100]
Megachile brevis	Wild	Adult	U.S.A.	2013	IAPV	11.7%	[127]
					SBV	70.6%	
					DWV	52.9%	
Megachile brevis	Wild	Adult	U.S.A.	2013	LSV	17.6%	[127]
Megachile rotundata	Wild	Adult	Canada	2013	SBV	56.0%	[100]
					DWV	48.0%	
	Artificial Infection		U.S.A.	2021	*C. bombi*	59.0%	[202]
				2020	*C. bombi*	-	[203]

Osmia bicornis	Wild	Adult	Georgia	2014	ABPV	-	[109]
			Germany		ABPV	-	
			Kyrgyzstan		ABPV	-	
			Georgia		IAPV	-	
			Germany		IAPV	-	
			Kyrgyzstan		IAPV	-	
			Georgia		BQCV	-	
			Germany		BQCV	-	
			Kyrgyzstan		BQCV	-	
			Georgia		SBV	-	
			Germany		SBV	-	
			Kyrgyzstan		SBV	-	
			Belgium		DWV[V]	100%	[37]
			Georgia		DWV	-	[109]
			Germany		DWV	-	
			Kyrgyzstan		DWV	-	
			Georgia		KBV	-	
			Germany		KBV	-	
			Kyrgyzstan		KBV	-	
			Georgia		SBPV	-	
			Germany		SBPV	-	
			Kyrgyzstan		SBPV	-	
			Belgium	2012	LSV[V]	100%	[84]
					*Am*FV[V]	100%	
					N. ceranae[V]	100%	
					C. bombi	100%	
					S. apis	33.3%	
					S. melliferum	66.7%	
					Ascosphaera spp.[V]	100%	
					Apicystis bombi[V]	100%	
Osmia cornuta	Wild	Adult	Belgium	2012	BQCV[W]	100%	[84]
	Artificial Infection		Italy	2014	DWV*	3.43%	[40]
	Wild		Georgia		DWV	100%	[109]
			Belgium	2012	LSV[W]	100%	[84]
					LSV*	100%	[227]
					*Am*FV[W]	100%	[84]
			Belgium	2012	*N. ceranae*[W]	100%	
					Ascoshpaera spp.[W]	100%	
					Apicystis bombi [W]	100%	
Osmia lignaria	Artificial Infection	Adult	U.S.A.	2021	*C. bombi*	29.0%	[202]
				2020		-	[203]
				2020	*C. mellificae*	-	[203]
**WASP**							
Bembicidae							
Bembix spp.	Wild	Adult	U.S.A.	2005	BQCV[X]	100%	[38]
				2005	DWV[X]	100%	
Cryptinae							
Xanthocryptus novozealandicus	Wild	Adult	New Zealand	2019	DWV	100%	[91]
Pompilidae							
Sphictostethus nitidus	Wild	Adult	New Zealand	2019	DWV	100%	[91]
Vespidae							
Ancistrocerus auctus	Wild	Adult	France	2021	ABPV[Y]	100%	[100]
					SBV[Y]	100%	
Dolichovespula sylvestris	Wild	Adult	U.S.A.	2008	*Wolbachia spp.*	100%	[128]
Polybia scutellaris	Wild	Adult	Argentina	2010	*N. ceranae*	100%	[163]
Polistes spp.	Wild	Adult	France	2021	ABPV[Z]	100%	[100]
					BQCV[Z]	100%	
					DWV[Z]	100%	
Polistes arifer	Wild	Adult	Hawaii	2015	DWV	45.0%	[222]
Polistes chinensis	Wild	Adult	New Zealand	2019	DWV	-	[91]
					Moku virus*	-	[91]
Polistes domicula	Wild	Adult	France	2021	ABPV	20.0%	[100]
					BQCV	20.0%	
					SBV	20.0%	
					DWV	20.0%	
Polistes fuscatus	Wild	Adult	U.S.A.	2005	DWV	16.7%	[38]
Polistes humilis	Wild	Adult	New Zealand	2019	Moku virus	-	[91]

Polistes metricus	Wild	Adult	U.S.A.	2005	BQCV[1]	25.0%	[38]
					SBV[1,2]	50.0%	
					DWV[1]	50.0%	
Polistes ninpha	Wild	Adult	France	2021	ABPV	100%	[100]
Vespa crabro	Wild	Adult	Italy	2016	DWV*	100%	[45]
			Spain	2016	*N. ceranae*	3.20%	[228]
					N. thompsoni	9.70%	
					C. bombi	12.9%	
					C. mellificae	3.2%	
					L. passim	16.1%	
					A. bombi	29.0%	
Vespa velutina nigrithorax	Wild	Adult	China	2011	IAPV*	100%	[48]
			Italy	2017	BQCV*	50.0%	[46]
					DWV*	75.0%	[47]
				2018		100%	[229]
					KBV*	8.34%	[46]
			Spain	2016	*N. ceranae*	2.50%	[228]
					N. thompsoni	11.4%	
					C. bombi	17.7%	
					C. mellificae	8.90%	
					L. passim	3.80%	
					A. bombi	17.7%	
			Belgium	2016	Moku virus	100%	[151]
Vespula spp.	Wild	Adult	U.S.A.	2011	BQCV	29.0%	[93]
					SBV	14.0%	
					DWV	57.0%	
Vespula germanica	Wild	Adult	United Kingdom	2018	ABPV	31.3%	[94]
			France		IAPV	3.23%	
			New Zealand		DWV	24.2%	
			Germany		DWV	9.68%	
			U.S.A.	2008	DWV	25.0%	[128]
			New Zealand	2019	DWV*[3]	100%	[91]
				2018	KBV	15.1%	[94]
				2019	KBV[3]	100%	[91]
			United Kingdom	2018	KBV	6.25%	[94]
			New Zealand	2019	Moku virus *[3]	100%	[91]
			U.S.A.	2008	*Wolbachia* spp.	25.0%	[128]
					Ascosphaera spp.	25.0%	
Vespula pensylvanica	Wild	Adult	Hawaii	2012	DWV	100%	[150]
					DWV	-	[130]
					Moku virus	-	
					Moku virus	100%	[150]
Vespula vulgaris	Wild	Adult	U.S.A.	2005	IAPV[4]	41.7%	[38]
					BQCV[4,5]	66.7%	[38]
				2008	BQCV	2.10%	[128]
				2005	SBV[4]	33.3%	[38]
				2008	SBV	2.10%	[128]
				2005	DWV[4,5]	91.7%	[38]
				2008	DWV	29.2%	[128]
			New Zealand	2019	DWV*		[91]
					KBV*	-	
		Larvae		2016	KBV	100%	[95]
	Artificial Infection				KBV	100%	
	Wild	Adult		2019	Moku virus*	-	[91]
		Larvae		2016	Moku virus	100%	[95]
	Artificial Infection				Moku virus	100%	
	Wild	Adult	U.S.A.	2008	Microsporidia	15.5%	[128]
					Wolbachia spp.	35.4%	
					Ascosphaera spp.	54.2%	
<u>Scolidae</u>							
Scolia flavifrons	Wild	Adult	France	2021	ABPV[6]	50.0%	[100]
					BQCV[6]	100%	
					SBV[6]	50.0%	
					DWV[6]	50.0%	
**ANT**							
<u>Formicidae</u>							
Anoplolepis gracilipes	Wild	Adult	Australia	2016	BQCV	-	[51]
			Japan	2019	DWV	3.40%	[230]
			Solomon Island		DWV	100%	
			Vanatu		DWV	50.0%	

Camponotus spp.	Wild	Adult	U.S.A.	2011	IAPV	6.00%	[93]
					BQCV	24.0%	
					SBV	24.0%	
					DWV	6.00%	
Camponotus vagus	Wild	Adult	France	2008	CBPV*	100%	[118]
					CBPV*	10.0%	
Formica rufa	Wild	Adult	France	2008	CBPV*	10.0%	[118]
Linepithema humile	Wild	Adult	New Zealand	2015	ABPV	-	[49]
					IAPV	-	
				2013	DWV*	7.00%	[50]
				2019	DWV*	-	[91]
					KBV*	-	[91]
				2015	KBV	-	[49]
				2019	Moku virus*	-	[91]
Paratrechina longicornis	Wild	Adult	Japan	2019	DWV	15.4%	[230]
			Taiwan		DWV	4.60%	
			Malaysia		DWV	13.6%	
			China		DWV	26.7%	
			Fiji		DWV	33.3%	
Pheidole megacephala	Wild	Adult	Hawaii	2012	DWV	-	[130]
Tapinoma melanocephalum	Wild	Adult	Hawaii	2012	DWV	-	[130]
Myrmicinae							
Myrmica rubra	Artificial Infection	Adult	Switzerland	2019	DWV	-	[231]

Legend: Bold and underlined taxon indicates the taxonomic group; Underlined taxon indicates the family; -: no calculated; *: replicative virus; superscript "A"-"Z" and "1"-"6": indicate the coinfection reported in the same study; ABPV: Acute Bee Paralysis Virus; IAPV: Israeli Acute Paralysis Virus; BQCV: Black Queen Cell Virus; SBV: Sacbrood Virus; DWV: Deforming Wing Virus; LSV; Lake Sani Virus; *Am*FV: *Apis mellifera* Filamentous Virus; KBV: Kashmir Bee Virus; SBPV: Slow Bee Paralysis Virus; CBPV: Chronic Bee Paralysis Virus; VdMLV: *Varroa destructor* Macula-like Virus.

Table S2. Bee pathogen spillover and prevalence identified in arthropod hosts, of which are reported condition, stage, geographical are and year.

Host			Geographic area	Year	Pathogen	Prevalence (%)	Reference
Taxon	Condition	Stage					
**FLY**							
Calliphoridae							
Undetermined Calliphoridae	Wild	Adult	U.S.A.	2011	DWV	25.0%	[93]
Muscidae							
Musca spp.	Wild	Adult	U.S.A.	2011	DWV	50.0%	[93]
Syrphidae							
Episyrphus balteatus	Wild	Adult	U.S.A.	2008	*Wolbachia* spp.	76.9%	[128]
					Ascosphaera spp.	69.2%	
Eristalis arbustorum	Wild	Adult	United Kingdom	2016	BQCV	10.0%	[129]
					SBV	5.00%	
					DWV	5.00%	
			U.S.A.	2008	*Wolbachia* spp.	72.7%	[128]
					Ascosphaera spp.	90.9%	
Eristalis tenax	Wild	Adult	United Kingdom	2016	BQCV	10.0%	[129]
					SBV	20.0%	
			U.S.A.	2008	Microsporidia	9.10%	[128]
					Wolbachia spp.	45.5%	
					Ascosphaera spp.	36.42%	
Rhingia campestris	Wild	Adult	U.S.A.	2008	*Wolbachia* spp.	41.7%	[128]
					Ascosphaera spp.	16.7%	
Tipulidae							
Leptotarus spp.	Wild	Adult	New Zealand	2019	DWV	-	[91]
**BEETLE**							
Coccinellidae							
Harmonia axyridis	Wild	Adult	U.S.A.	2011	DWV	100%	[93]
Curculionidae							
Scolopterus penicillatus	Wild	Adult	New Zealand	2019	DWV	100%	[91]
Nitidulidae							
Aethina tumida	Wild	Adult	U.S.A.	2011	IAPV	78.0%	[93]
					BQCV	5.00%	
			Hawaii	2012	BQCV	-	[130]
	Laboratory		U.S.A.	2008	SBV	33.4%	[82]
	Wild			2011	SBV	11.0%	[93]
			Hawaii	2012	SBV	-	[130]
			U.S.A.	2017	DWV*[7]	70.0%	[164]
				2011	DWV	72.0%	[93]
			Hawaii	2012	DWV	-	[130]
	Artificial Infection	Adult	U.S.A.	2007	DWV*	97.0%	[232]
				2019	DWV		[86]
	Wild	Adult	U.S.A.	2017	KBV*[7]	20.0%	[164]
				2011	KBV	44.0%	[93]
			Mexico	2018	*Am*FV[8,9]	100%	[148]
			U.S.A.	2017	*N. ceranae*	70.0%	[164]
		Larvae	Mexico	2018	*N. ceranae*[9]	25.0%	[148]
		Adult	U.S.A.	2017	*L. passim*[7]	40.0%	[164]
			Mexico	2018	*L. passim*[8,9]	83.4%	[148]
			U.S.A.	2017	*C. mellificae*[7]	30.0%	[164]
			Mexico	2018	*C. bombi*[8]	50.0%	[148]
					Ascosphaera spp.[8]	16.7%	
Tenebrionidae							
Undetermined Tenebrionidae	Wild	Adult	U.S.A.	2011	DWV	100%	[93]
**ROACH**							
Blattidea							
Celatoblatta spp.	Wild	Adult	New Zealand	2019	DWV*	-	[91]
					KBV	-	
					Moku virus	-	
Maoriblatta spp.	Wild	Adult	New Zealand	2019	DWV	-	[91]
					Moku virus	-	
Ectobiidae							
Blattella germanica	Wild	Adult	U.S.A.	2011	BQCV	50.0%	[93]
					SBV	75.0%	
					DWV	100%	

**EARWIG**							
Forficulidae							
Forficula auricularia	Wild	Adult	U.S.A.	2011	IAPV	40.0%	[93]
					BQCV	60.0%	
					SBV	50.0%	
					DWV	100%	
			New Zealand	2019	DWV	-	[91]
			U.S.A.	2011	KBV	20.0%	[93]
			New Zealand	2019	KBV	-	[91]
**BUG**							
Pentatomidae							
Halymorpha halys	Wild	Adult	U.S.A.	2011	DWV	100%	[93]
**CRICKET**							
Trigonidiidae							
Bobilla spp.	Wild	Adult	New Zealand	2019	DWV	-	[91]
					KBV*	-	
**BUTTERFLY**							
Undetermined Lepidoptera	Wild	Adult	U.S.A.	2011	IAPV	9.00%	[93]
					BQCV	9.00%	
					SBV	9.00%	
					DWV	27.0%	
Papilionidae							
Undetermined Papilionoidae	Wild	Adult	U.S.A.	2011	DWV	100%	[93]
**MOTH**							
Pyralidae							
Galleria melonella	Wild	Larvae	U.S.A.	2011	IAPV	14.0%	[93]
					BQCV	33.0%	
					SBV	29.0%	
					DWV	71.0%	
					KBV	10.0%	
**SPIDER**							
Undetermined Aranae	Wild	Adult	U.S.A.	2011	IAPV	10.0%	[93]
					BQCV	10.0%	
					SBV	10.0%	
					DWV	80.0%	
Undetermined Opiliones	Wild	Adult	U.S.A.	2011	IAPV	10.0%	[93]
					BQCV	10.0%	
					DWV	10.0%	
Salticidae							
Helpis minitabunda	Wild	Adult	New Zealand	2019	DWV	100%	[91]
					Moku virus	100%	
Theridiidae							
Steatoda capensis	Wild	Adult	New Zealand	2019	KBV	-	[91]
					Moku virus*	-	

Legend: Bold and underlined taxon indicates the taxonomic group; Underlined taxon indicates the family; -: no calculated; *: replicative virus; superscript "7"-"9": indicate the coinfection reported in the same study -: no calculated; IAPV: Israeli Acute Paralysis Virus; BQCV: Black Queen Cell Virus; SBV: Sacbrood Virus; DWV: Deforming Wing Virus; LSV; Lake Sani Virus; *Am*FV: *Apis mellifera* Filamentous Virus; KBV: Kashmir Bee Virus.
